# Population stratification may bias analysis of PGC-1α as a modifier of age at Huntington disease motor onset

**DOI:** 10.1007/s00439-012-1205-z

**Published:** 2012-07-25

**Authors:** Eliana Marisa Ramos, Jeanne C. Latourelle, Ji-Hyun Lee, Tammy Gillis, Jayalakshmi S. Mysore, Ferdinando Squitieri, Alba Di Pardo, Stefano Di Donato, Michael R. Hayden, Patrick J. Morrison, Martha Nance, Christopher A. Ross, Russell L. Margolis, Estrella Gomez-Tortosa, Carmen Ayuso, Oksana Suchowersky, Ronald J. Trent, Elizabeth McCusker, Andrea Novelletto, Marina Frontali, Randi Jones, Tetsuo Ashizawa, Samuel Frank, Marie-Helene Saint-Hilaire, Steven M. Hersch, Herminia D. Rosas, Diane Lucente, Madaline B. Harrison, Andrea Zanko, Karen Marder, James F. Gusella, Jong-Min Lee, Isabel Alonso, Jorge Sequeiros, Richard H. Myers, Marcy E. MacDonald

**Affiliations:** 1 Center for Human Genetic Research, Massachusetts General Hospital, Simches Research Building, Room 5414, 185 Cambridge Street, Boston, MA 02114 USA; 2 UnIGENe, IBMC, Institute for Molecular and Cell Biology, University of Porto, Porto, Portugal; 3 Department of Neurology, Boston University School of Medicine, Boston, MA 02118 USA; 4 Centre for Neurogenetics and Rare Diseases, IRCCS Neuromed, 86077 Pozzilli (IS), Italy; 5 Fondazione IRCCS Istituto Neurologico Carlo Besta, via Celoria 11 20133, Milan, Italy; 6 Center for Molecular Medicine and Therapeutics, University of British Columbia, Vancouver, BC V5Z 4H4 Canada; 7 Regional Medical Genetics Centre, Belfast HSC Trust, Belfast, BT9 7AB UK; 8 University of Ulster, Cromore Road, Coleraine, BT52 15A UK; 9 Hennepin County Medical Center, 701 Park Avenue, Minneapolis, MN 55415 USA; 10 Department of Psychiatry and Behavioral Sciences, Johns Hopkins University, Baltimore, MD 21287 USA; 11 Department of Neurology, Fundación Jiménez Diaz, 28040 Madrid, Spain; 12 Department of Genetics, IIS, Fundación Jiménez Diaz, CIBERER, 28040 Madrid, Spain; 13 Departments of Medicine and Medical Genetics, University of Alberta, Edmonton, AB T6G 2B7 Canada; 14 Sydney Medical School, University of Sydney, Sydney, NSW 2006 Australia; 15 Department of Neurology, Westmead Hospital, Westmead Sydney, NSW 2145 Australia; 16 Department of Biology, University Tor Vergata, 00133 Rome, Italy; 17 Institute of Translational Pharmacology, CNR 00133 Rome, Italy; 18 Department of Neurology, Emory University, Atlanta, GA 30329 USA; 19 Department of Neurology, University of Florida, Gainesville, FL 32610 USA; 20 MIND, Massachusetts General Hospital, 114 16th Street, Charlestown, MA 02129 USA; 21 Department of Neurology, University of Virginia, Charlottesville, VA 22908 USA; 22 Department of Pediatrics, University of California, San Francisco, CA 94143 USA; 23 College of Physicians and Surgeons, Columbia University, New York, NY 10032 USA; 24 Harvard Medical School and Program in Medical and Population Genetics, Broad Institute of Harvard and Massachusetts Institute of Technology, Cambridge, MA 02142 USA; 25 CGPP, IBMC, Institute for Molecular and Cell Biology, University of Porto, Porto, Portugal; 26 ICBAS, Instituto de Ciências Biomédicas Abel Salazar, University of Porto, Porto, Portugal

## Abstract

Huntington’s disease (HD) is an inherited neurodegenerative disorder characterized by motor, cognitive and behavioral disturbances, caused by the expansion of a CAG trinucleotide repeat in the HD gene. The CAG allele size is the major determinant of age at onset (AO) of motor symptoms, although the remaining variance in AO is highly heritable. The rs7665116 SNP in *PPARGC1A*, encoding the mitochondrial regulator PGC-1α, has been reported to be a significant modifier of AO in three European HD cohorts, perhaps due to affected cases from Italy. We attempted to replicate these findings in a large collection of (1,727) HD patient DNA samples of European origin. In the entire cohort, rs7665116 showed a significant effect in the dominant model (*p* value = 0.008) and the additive model (*p* value = 0.009). However, when examined by origin, cases of Southern European origin had an increased rs7665116 minor allele frequency (MAF), consistent with this being an ancestry-tagging SNP. The Southern European cases, despite similar mean CAG allele size, had a significantly older mean AO (*p* < 0.001), suggesting population-dependent phenotype stratification. When the generalized estimating equations models were adjusted for ancestry, the effect of the rs7665116 genotype on AO decreased dramatically. Our results do not support rs7665116 as a modifier of AO of motor symptoms, as we found evidence for a dramatic effect of phenotypic (AO) and genotypic (MAF) stratification among European cohorts that was not considered in previously reported association studies. A significantly older AO in Southern Europe may reflect population differences in genetic or environmental factors that warrant further investigation.

## Introduction

Huntington’s disease (HD) is a neurodegenerative disorder with classic symptoms that include progressive chorea, motor deficits, cognitive changes and dementia. Age at onset (AO) of the overt symptoms is highly variable: while some individuals show signs in the first decade, others remain asymptomatic even after 60 years of age, though in most cases death follows on average about 17 years after the first symptoms. HD is inherited as an autosomal dominant trait and is caused by the expansion of an unstable CAG repeat, in the first exon of the HD gene (now called *HTT*), on chromosome 4p16.3 (The Huntington’s Disease Collaborative Research Group 1993), resulting in an expanded polyglutamine tract in the huntingtin protein.

The major determinant of AO in HD is the size of the expanded CAG repeat allele (Lee et al. 2012), such that the longer the repeat the earlier the onset of clinical symptoms, though most HD cases occur in adulthood with about 40–45 CAG repeats. Repeat length alone explains about 70 % of the variability in onset age (Duyao et al. 1993). The remaining variance in AO (residual AO) is highly heritable but remains unexplained. Nonetheless, recent genetic studies have nominated about 20 loci that may modify AO or progression of HD. However, many of the specific polymorphisms assessed in multiple studies have failed to be replicated, including attractive biological candidate genes such as glutamate receptor, ionotropic kainate 2 (*GRIK2*), apolipoprotein E (*APOE*) and brain-derived neurotrophic factor (*BDNF*) (Rubinsztein et al. 1997; Metzger et al. 2006; Panas et al. 1999; Alberch et al. 2005; Di Maria et al. 2006).

One biologically compelling candidate thought to be involved in HD pathogenesis is *PPARGC1A*, localized at 4p15.1-2, which encodes peroxisome proliferator-activated receptor γ coactivator 1α (PGC-1α), a transcriptional regulator of adaptive thermogenesis (Puigserver et al. 1998) and mitochondrial respiration and oxidative stress (Puigserver and Spiegelman 2003; St-Pierre et al. 2006). The lack of PGC-1α expression produces an HD-like phenotype in mice (Lin et al. 2004; Leone et al. 2005) and mutant, but not wild type, huntingtin down-regulates the expression of PGC-1α and its target genes (Cui et al. 2006; Lin et al. 2004; Weydt et al. 2006). Moreover, three recent studies, with DNA samples from European HD patients, mainly from Italy and Germany, have reported an association of PGC-1α with AO of HD symptoms (Che et al. 2011; Weydt et al. 2009; Taherzadeh-Fard et al. 2009). Although different sets of *PPARGC1A* SNPs were included in these studies, one polymorphism (rs7665116), located at the 3′-end region of intron 2, was associated with a later AO in all three studies, displaying a significant effect both in an additive and dominant model. Following these results, it was reported that polymorphisms in PGC-1α downstream target genes, namely nuclear respiratory factor 1 (*NRF1*) and mitochondrial transcription factor A (*TFAM*), may influence the AO in HD (Taherzadeh-Fard et al. 2011). Given that results based on analyses of the *PPARGC1A* rs7665116 SNP are motivating a broader range of research into the functional basis of the effect, the aim of the present study was to attempt to replicate the association of this SNP with AO, in a much larger cohort of 1,727 HD patients of different European populations.

## Methods

### Subjects

We analyzed 1,929 HD patients with known AO of overt motor symptoms. The DNA samples were from subjects involved in long-term genetic studies from collaborating investigators (HD-MAPS), the HD observational study COHORT and from the Harvard Tissue Resource Center Bank (McLean’s Hospital, Belmont MA) and the National Neurological Research Bank (VAMC Wadsworth Division, Los Angeles CA). These studies included related individuals [from 1,676 different families defined either based on the likelihood of genetic similarity from genome-wide genotyping information (Western European samples) or membership in nuclear (parents and children) families (Southern European samples)]. Of these, 934 were self-reported as originally from Southern European countries (263 from Portugal, 664 from Italy, 5 from Spain and 2 from Greece), the rest of the cases had unconfirmed or no geographical origin data. 1,020 of these were genotyped using the GeneChip Human Mapping 500K Array Set (Affymetrix) at the Broad Institute of Harvard and MIT as part of a genome-wide scan for HD genetic modifiers.

### Genotyping

The HD CAG repeat length was determined by a polymerase chain reaction (PCR) amplification assay, using fluorescently labeled primers, as previously described (Warner et al. 1993). The size of the fragments was determined using the ABI PRISM 3730*xl* automated DNA Sequencer (Applied Biosystems, Foster City, CA, USA) and GeneMapper version 3.7 software. A set of HD CAG alleles, determined by DNA sequencing, were used as standards. Genotyping of the PGC-1α polymorphism (rs7665116) was performed by real-time PCR using the commercially available Taqman Genotyping probe (Applied Biosystems, Foster City, CA, USA) carried out on the LightCycler^®^ 480 (Roche Diagnostics, Mannheim), following manufacturer’s instructions.

### Statistics

For the 1,020 samples with whole-genome genotyping, PCA was carried out using PLINK v1.05 (http://pngu.mgh.harvard.edu/Purcell/plink/) (Purcell et al. 2007) in order to determine the genetic ancestry of these individuals. Briefly, genotypes of HD samples were combined with HapMap Phase 2 data (CEPH, Yoruba, Han-Chinese and Japanese populations) for pairwise IBD estimation and subsequent IBS clustering.

To assess differences in the mean motor AO among Western and Southern European samples, we used the general estimating equation (GEE), thereby adjusting for related samples. Multivariate analyses were generated using GEE to assess the effect of the rs7655116 SNP at the PGC-1α gene with HD residual motor onset, adjusting for familial correlation. Residual motor onsets were computed as the difference between the observed and expected age of onset and were standardized to a mean of zero and standard deviation of one. The weighted GEE was computed assuming an independent correlation structure and using the robust estimator of the variance to account for familial relationships. All statistical analyses were performed using PASW Statistics (version 18).

## Results

We genotyped a collection of 1,929 HD DNA samples, with known HD CAG allele sizes and known age at onset of motor symptoms, for the *PPARGC1A* rs7665116 polymorphism. The observed genotype frequency of this SNP was in Hardy–Weinberg equilibrium. Since, in two of the previous reports, the association with AO was primarily observed in HD patients of Italian ancestry (Che et al. 2011; Weydt et al. 2009); we split our large cohort by ancestry into either Southern European or Western European HD cases. The Southern European HD cases (*n* = 934) consisted of self-reported Portuguese (*n* = 263), Italian (*n* = 664), Spanish (*n* = 5) and Greek (*n* = 2) HD cases. The Western European HD cases were chosen from amongst another 1,020 HD patients by use of principal component analysis (PCA) on available whole-genome genotyping data, to infer their genetic background. The first principal component (PC1) distinguished Africans from non-Africans and the second principal component (PC2) distinguished Africans and Europeans from Asians (data not shown), and allowed us to exclude from our analysis the few samples who had significant contribution of either Asian or African ancestry. Among the remaining (*n* = 952) European cases, the Western European cluster (*n* = 793) was defined by overlap with the US Northern-Western European origin CEPH (HapMap) cluster, and consisted mainly of persons with self-reported North-American origin (Canada and US) as well as French and Irish. Thus, we had a total of 1,727 HD patients with assigned ancestry; 934 Southern European and 793 Western European (Table 1).

**Table 1:** Genetic and clinical data among European cases of Western and Southern origin

Origin of samples	36-87 HD CAG range				40-53 HD CAG range			
	Number of samples	Mean HD CAG (median)	Mean Motor Onset (median)	rs7665116 MAF	Number of samples	Mean HD CAG (median)	Mean Motor Onset (median)	rs7665116 MAF
Southern European^a^	934	44.29 (43)	47.34 (48)	0.169	879	43.73 (43)	48.04 (48)	0.173
Western European^b^	793	44.78 (44)	43.20 (43)	0.119	749	44.32 (44)	43.83 (43)	0.118
Total European	1,727	44.52 (43)	45.44 (45)	0.146	1,628	44.00 (43)	46.10 (46)	0.148

^**a**^Self-reported

^**b**^Genetic background confirmed

Remarkably, analysis of the clinical data for these 1,727 HD cases, which had CAG alleles ranging from 36 to 87 repeats, revealed that the self-reported Southern Europeans (*n* = 934) had significantly later onset of motor HD symptoms *(*p < 0.001), by 4–5 years, compared to the Western European (*n* = 793), though the means/medians for HD CAG repeat length were similar in both groups (Table 1). Furthermore, the observed rs7665116 genotypes for samples from the Southern European countries revealed higher minor allele frequency (MAF) (~17 %) when compared to the Western European set (~12 %) (Table 1). These findings, together with the striking differences in the MAF of this polymorphism among the different HapMap populations, strongly suggested that population stratification might increase type I errors in the AO association analysis.

Finally, we have recently shown that the non-normal distribution of CAG allele size (and AO) also introduces error in conventional statistical analysis (Lee et al. 2012). Even a single CAG outlier sample, with a very long CAG repeat and extremely young age at onset relative to all others, can have a profound effect on the final result when testing for the effects of potential genetic modifiers (Lee et al. 2012). Therefore, as a final filter, we chose only the Southern and Western European HD cases with CAG alleles in the 40- to 53-repeat range, shown previously to yield a statistically well-behaved data set that conforms to the fundamental assumptions of linear regression analysis (constant variance and normally distributed error) (Lee et al. 2012).

**Fig. 1:**
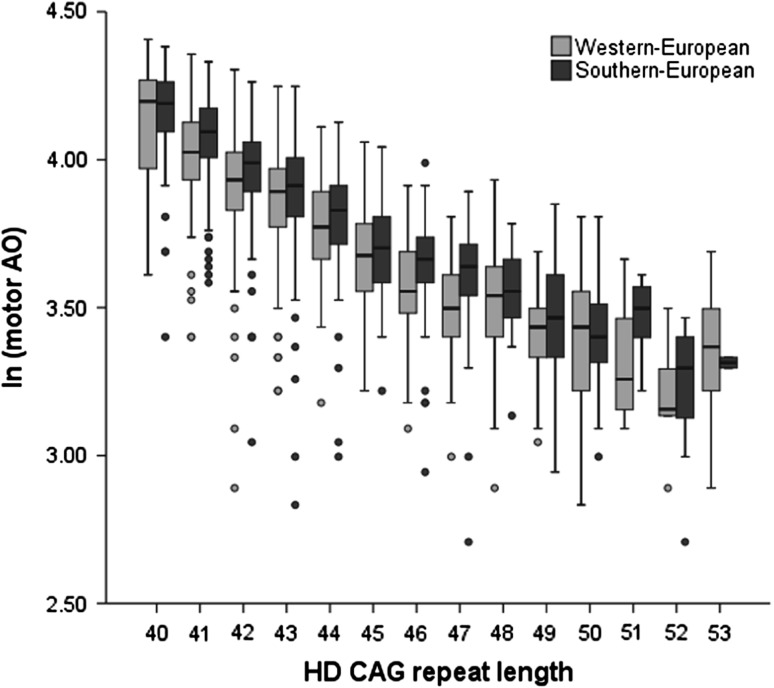
Variance of age at motor onset for HD cases of Western and Southern European origin. A *box plot* depicting the relationship of the natural log-transformed age at onset of motor symptoms to the expanded CAG allele size, for patients in the 40–53 CAG range, illustrating that self-reported Southern Europeans had an older age at onset across the spectrum of allele sizes. *Circles* are outliers defined by a standard quartile method (outside of 1.5 times interquartile range), some of which could reflect errors in the motor AO data while others may represent true biological outliers

As summarized in Table 1, these filtering steps yielded a total of 879 self-reported Southern European cases from 823 families and 749 Western European cases (matched by use of PCA) from 620 different families, with CAG repeats ranging from 40 to 53 repeats. Notably, as observed for the larger set of HD patients with a broader CAG repeat range, in this final set of 1,628 patients the self-reported Southern Europeans had a significantly older mean age at onset of motor symptoms than Western Europeans (Table 1), which was observed across the spectrum of CAG allele sizes (Fig. 1), despite a similar mean/median HD CAG repeat size (Table 1). Furthermore, the rs7665116 MAF was higher in the former relative to the latter Western European patients (Table 1).

Using this final set of 1,628 HD patients, we then performed analysis to determine whether rs7665116 may contribute to variance in HD motor onset not explained by the length of the expanded CAG repeat. In order to adjust for familial relationships, the effect of the rs7665116 on residual motor onset was calculated using generalized estimating equations (GEE). In the unadjusted analysis, a significant association with later residual AO was observed for both the additive genetic model (β = 0.090, *p* value = 0.009) and the dominant model (β = 0.113, *p* value = 0.008) (Table 2). However, adjusting the analysis for ancestry (Southern vs. Western European), in both the additive and the dominant models, produced a striking impact on the effect sizes (β decreased by ~25 %) and the *p* values (*p* increased ~4×) (Table 2), thereby revealing that population stratification is a large contributor to an apparent rs7665116 association.

**Table 2:** Multivariate correlation of rs7665116 with residual age at motor onset

Model	Standardized coefficient	Standard error	95 % confidence limits		*p* value*
*Additive*					
PGC1α rs7665116					
T–T vs. T–C vs. C–C	**0.090**	0.0348	0.022	0.158	**0.009**
PGC1α rs7665116 + ancestry					
T–T vs. T–C vs. C–C	**0.069**	0.0345	0.001	0.136	**0.047**
WE vs. SE	0.208	0.0416	0.127	0.290	<0.001
*Dominant*					
PGC1α rs7665116					
T–T vs. T–C + C–C	**0.113**	0.0424	0.029	0.196	**0.008**
PGC1α rs7665116 + ancestry					
T–T vs. T–C + C–C	**0.089**	0.0418	0.007	0.171	**0.033**
WE vs. SE	0.208	0.0415	0.127	0.289	<0.001

* p values were derived using GEE to account for familial relationships

## Discussion

Previous studies have reported the presence of a common polymorphism in *PPARGC1A* (rs7665116) that is associated with a delay in AO of HD motor symptoms in three European HD cohorts (Che et al. 2011; Taherzadeh-Fard et al. 2009; Weydt et al. 2009), primarily contributed by patients from Italy. Our study, which involved a larger collection of HD cases, did not provide strong evidence for this SNP, and therefore, for PGC-1α as a modifier of HD motor onset, but did strongly support further investigation of the factors that contribute to the striking differences in AO of motor symptoms in ‘Southern Europeans’.

The results of our study expose genetic ancestry as a critical factor in HD association studies. It is expected that a disease-associated polymorphism may have varying effects in different populations, but the variation in minor allele frequency, across different genetic backgrounds related to ancestry may also be critical and should be taken into account in genetic association analysis. Cases that are poorly matched for genetic background may lead to false positives in association studies. It is important to control for ancestry by use of PCA or related methods, even in apparently close related populations such as Europeans (Novembre et al. 2008) where there is strong evidence of recent population selection that has led to intra-European variation in allele frequency (Price et al. 2008). This is particularly important in studies using the CEPH-CEU panel as controls since the genetic matching to different populations may differ considerably in different countries (Lao et al. 2008). It is difficult to accurately infer ancestry in candidate gene association studies, leading to imperfect correction for stratification. However, with increasing availability of genome-wide datasets, the assessment of population structure should become a common procedure for candidate association studies.

Our study points out the dramatic effect that population stratification can have in testing a candidate gene for an association with disease phenotype. We found that the variation in rs7665116 minor allele frequency could lead to a false positive, if genetic ancestry is not corrected for in the analysis. The Southern European cases seem to be different genetically and clinically, in terms of age at diagnosis of motor symptoms, from other European samples. By adjusting for ancestry, we observed striking effects on both the P values (increased ~4×) and effect sizes (decreased by ~25 %). Even though the post-adjustment *p* values remained nominally significant, the dramatic reduction in significance occasioned by considering ancestry does not lend confidence to its being a true effect and rather suggests that it is due to insufficiently rigorous ancestry categorization of the ‘Southern European’ set, which was based solely on self-reporting rather than unbiased genome-wide genotyping data. Consistent with this interpretation, while this manuscript was under review, an article by Soyal et al. (2012) reported that no association of rs7651166 with AO to first symptom (not necessarily motor) was found in an European cohort of 1,706 HD patients whose MAF for this SNP closely resembled the MAF that we report for the Western European samples in our study.

A remarkable finding from our analysis is the strong evidence of later motor onset for HD patients originally from Southern European countries (Portugal and Italy), which have a reported onset of motor symptoms that is 4–5 years later than that of HD patients from other European regions, despite similar mean/median CAG allele size. This striking difference may reflect population differences elsewhere in the genome, since extensive genome-wide SNP analyses have shown that even though European populations share much of their genetic background, they also exhibit a notable degree of non-sharing (ancestry) (Lao et al. 2008; Novembre et al. 2008). It’s important to note that in addition to genetic background differences, a number of other factors may contribute to the difference in age at onset. One possibility is environmental influences, for example, differences related to lifestyle, or perhaps types of medication. A meta-analysis study has found evidence that people who adhere to a Mediterranean Diet appear to have a reduced risk of developing Parkinson’s and Alzheimer’s disease (Sofi et al. 2008), and altered Alzheimer’s disease course (Scarmeas et al. 2007). A recent observational study of HD in Europe has shown that Southern European (and Polish) clinicians prescribed anti-dyskinetic medication more frequently than clinicians in other European regions (Orth et al. 2010). Another environment related factor that is likely to contribute are the criteria and procedures for diagnosing HD, which may differ in different cultures. It is now important to understand which of many potential population-specific genetic and/or environmental factors are associated with later reported AO of motor symptoms in Southern Europeans.

## Conclusion

The results of our study do not provide strong evidence for *PPARGC1A* SNP rs7665116, and therefore, for PGC-1α, as a modifier of age at onset of HD motor symptoms. However, we have found evidence of a significantly later age at onset of motor symptoms in Southern European countries, which may reflect genetic effects and/or environmental (lifestyle, diagnosis) factors that should be further explored. Our data strongly illustrate the false contribution that population stratification may make in a candidate gene association study, while providing genetic evidence that the contribution of PGC-1α as a modifier of the disease process that leads to onset of HD motor symptoms may not be significant.

## Appendix: Huntington Study Group COHORT Investigators

### Steering Committee

University of Rochester, Rochester, NY: Ira Shoulson, MD (principal investigator), James Gusella, PhD (co–principal investigator), Tatiana Foroud (co-principal investigator), Irina Antonijevic, Dan van Kammen (CHDI Foundation Inc.).

### Publications and Data Use Committee

Tatiana Foroud (Chair), Ray Dorsey (Co-Chair), John Warner (CHDI), Joe Giuliano, Louise Vetter, Oksana Suchowersky, Christopher Beck, David Oakes.

### Participating Investigators and Coordinators

F. Marshall, University of Rochester;

K. Marder, S. Frucht, C. Moskowitz, R. Clouse, P. Wasserman, Columbia University Medical Center;

K. Shannon, J. Jaglin, Rush University Medical Center;

J. Jankovic, A. Palao, Baylor College of Medicine;

M. Harrison, University of Virginia;

C. Singer, M. Quesada, University of Miami;

S. Hersch, D. Rosas, K. Tanev, K. Malarick, Massachusetts General Hospital;

A. Colcher, University of Pennsylvania;

J. Sanchez-Ramos, University of South Florida;

S. Kostyk, Ohio State University;

J. Paulsen, University of Iowa;

J. Perlmutter, S. Tabbal, Washington University;

C. Ross, F. Nucifora, Johns Hopkins University;

R. Dubinsky, H. Dubinsky, University of Kansas Medical Center;

O. Suchowersky, M.L. Klimek, University of Calgary

R. Jones, C. Testa, S. Factor, Emory University School of Medicine;

D. Jennings, Institute for Neurological Disorders

J. Morgan, Medical College of Georgia;

D. Higgins, E. Mohlo, Albany Medical College;

J. Adams, The Centre for Addiction and Mental Health, Toronto, Canada;

S. Frank, M. Saint-Hilaire, M. Diggin, Boston University;

S. Furtado, University of Alberta

F. Walker, C. O’Neill, V. Hunt, Wake Forest University School of Medicine;

K. Quaid, Indiana University School of Medicine;

M. LeDoux, University of Tennessee Health Science Center;

L. Raymond, B. Leavitt, J. Decolongon, University of British Columbia, Canada;

S. Perlman, University of California, Los Angeles

J. Corey-Bloom, G. Peavy, J. Goldstein, University of California San Diego;

R. Kumar, Colorado Neurological Institute;

E. McCusker, J. Griffith, C. Loy, Westmead Hospital, NSW, Australia;

V. Wheelock, T. Tempkin, A. Martin, University of California Davis;

M. Nance, Hennepin County Medical Center;

U. Kang, University of Chicago;

W. Mallonee, G. Suter, Hereditary Neurological Disease Center, Kansas;

F. Revilla, M. Gartner University of Cincinnati/Cincinnati Children’s Hospital;

C. Drazinic, M.J. Fitzpatrick, University of Connecticut;

M. Panisset, Hôtel-Dieu Hôpital-CHUM, Canada;

K. Duff, University of Utah

B. Scott, Duke University Medical Center;

W. Weiner, B. Robottom, University of Maryland School of Medicine;

E. Chiu, O. Yastrubetskaya, A. Churchyard, St Vincent’s Aged Mental Health Service (SVAMHS) Melbourne, Australia;

T. J. Greenamyre, University of Pittsburgh;

P. Agarwal, Booth Gardner Parkinson’s Care Center, WA.

Biostatistics/Coordination Center: University of Rochester—D. Oakes, C. Beck, S. Robertson, K. Eaton, P. Lindsay, L. Deuel.

DNA Genotyping Center: M. MacDonald, Center for Human Genetic Research, Massachusetts General Hospital.

### Contributors

C. Hickey, University of Rochester; L. Muratori, E. Louis, Columbia University Medical Center; A. Leserman, N. Doucette, Ohio State University; E. Uc, R. Rodnitzky, S. Vik, University of Iowa, R. Davis, S. Dietrich, University of Virginia; V. Segro, D. Erickson, Colorado Neurological Institute; V. Hunt, Wake Forest University School of Medicine; N. Lucarelli, University of Pittsburgh; J. Broyles, University of Kansas Medical Center; J. DeLaRosa, University of Texas (Galveston); E. Louis, Columbia University Medical Center; P. Panegyres, Neurodegenerative Disorders Research, WA, AUS; A. Schmidt, S. Barton, Washington University; E. Sperin, C. Testa, Emory University; F. Thiede, University of California, Los Angeles; S. E. Zauber, M. Wesson, Indiana University; R. McInnis, Massachusetts General Hospital; C. Welsh, Johns Hopkins University; M. Wesson, Indiana University School of Medicine; A. Coleman, University of British Columbia, Canada.
